# COMBATTING LONELINESS: SERVICE USE AMONG RURAL FAMILY CAREGIVERS OF PERSONS WITH DEMENTIA

**DOI:** 10.1093/geroni/igz038.3249

**Published:** 2019-11-08

**Authors:** Emily K Hoyt, Karen A Roberto, Jyoti Savla

**Affiliations:** 1 Virginia Polytechnic Institute and State University, Blacksburg, Virginia, United States; 2 Virginia Tech, Blacksburg, Virginia, United States

## Abstract

According to a 2018 AARP study, 42% of unpaid caregivers experience loneliness. While findings across multiple studies suggest that caregivers experience loneliness either because they lack intimacy in close relationships (i.e., emotional loneliness) or they feel disconnected from their social network (i.e., social loneliness), little is known about how aspects of dementia caregiving influence loneliness, particularly among rural caregivers. The purpose of this study was to examine the association between in-home service use and caregivers experience with both types of loneliness. Eighty-eight co-residing dementia caregivers in rural Appalachia (Mean Age = 68 years; 91% White; 58% Spouses) completed telephone interviews that included questions about their use of formal services and perceptions of emotional and social loneliness. More than half (58%) of the caregivers accessed 1 to 4 formal services. Regression models revealed that caregivers who experienced greater social loneliness were more likely to access personal care services (p=0.013) and respite services (p=0.004) compared to caregivers who experienced less social loneliness. Further, caregivers who experienced greater emotional loneliness were also more likely to access personal care (p=0.028) and respite (p=0.039) services compared to caregivers who experienced lower emotional loneliness. These associations remained robust even after controlling for relationship to the PwD (spouse vs. non-spouse). Findings suggest that beyond assisting with the care of the PwD, the use of formal services may help family caregivers manage loneliness and relieve social isolation. Discussion will focus on the importance of service accessibility and use for the health and psychological well-being of rural family caregivers.

